# Case of a Woman Poisoned by Opium, with the Appearances on Dissection

**Published:** 1807-05

**Authors:** M. Lassus


### Case of a Woman poisoned by Opium, with the Appearances on Dissection. *By* M. Lassus.

A Woman, aged 60, who had for several years been subject to fits of melancholy, purchased thirty-six grains of opium, the whole of which she swallowed in the middle of the night. Five or six hours afterwards, she was found in a profound stupor, accompanied with stertorous respiration, as if labouring under apoplexy. She nevertheless for a few moments recovered some small degree of recollection; and it was during this interval, that we learned from her own confession, the circumstance above mentioned. She was immediately ordered a dose of ipecacuanha, which, however, produced no effect; she afterwards swallowed with much difficulty some spoonfuls of vinegar and water. I saw her at that time with M. Fourcroy. She was wholly unconscious, and evinced not the smallest appearance of sensibility. Her respiration was difficult and laborious, skin hot, pulse hard and full, the pupils of the eyes were much dilated, the joints flexible, and all the muscles in a state of complete relaxation. She expired without without experiencing the slightest convulsions, ten or eleven hours after swallowing the opium.

On opening the body, we found the internal coat of the stomach much inflamed, but without any marks of erosion. The inflammation extended throughout the small intestines, which were in many places in a gangrenous state, and of a greenish colour. In the cavity of the stomach were found about five or six spoonfuls of a turbid and reddish coloured fluid, which appeared to be the vinegar the patient had swallowed, intermingled with the mucus of the stomach and the ipecacuanha. The opium had been so completely dissolved, that not the least portion of it could be detected.

The small intestines appeared in a collapsed state, while the cæcum and the colon were inflated with air, and much distended. The abdominal and thoracic viscera were in their natural state. But we found in the gall-bladder three large calculi, which, from the report of her relations, had never, however, given her any distress. The blood contained in the cavities of the heart, and contiguous vessels, was blackish, and coagulated, as in other dead bodies. The brain displayed no marks of disease; its vessels were not preternaturally distended with blood; nor did any serosity appear in the ventricles. In a word, the seat of the disease seemed to be confined totally to the stomach and small intestines.

It follows from this case; first, that two large a dose of opium does not produce apoplexy, or, in other words, a congestion of blood in the vessels of the brain.

Second, that its activity is in proportion to the rapidity of its solution in the stomach; and that it excites inflammation in that organ, which speedily terminates in gangrene.

Third, that the vegetable acids, usually considered by practitioners as a specific in such cases, will only prove efficacious when the opium has been taken in a small quantity.

